# Effective Ransomware Detection Using Entropy Estimation of Files for Cloud Services

**DOI:** 10.3390/s23063023

**Published:** 2023-03-10

**Authors:** Kyungroul Lee, Jaehyuk Lee, Sun-Young Lee, Kangbin Yim

**Affiliations:** 1 Department of Information Security Engineering, Mokpo National University, Muan 58554, Republic of Korea; 2 Interdisciplinary Program of Information & Protection, Mokpo National University, Muan 58554, Republic of Korea; 3 Department of Information Security Engineering, Soonchunhyang University, Asan 31538, Republic of Korea

**Keywords:** cloud service, entropy, malicious code, ransomware

## Abstract

A variety of data-based services such as cloud services and big data-based services have emerged in recent times. These services store data and derive the value of the data. The reliability and integrity of the data must be ensured. Unfortunately, attackers have taken valuable data as hostage for money in attacks called ransomware. It is difficult to recover original data from files in systems infected by ransomware because they are encrypted and cannot be accessed without keys. There are cloud services to backup data; however, encrypted files are synchronized with the cloud service. Therefore, the original file cannot be restored even from the cloud when the victim systems are infected. Therefore, in this paper, we propose a method to effectively detect ransomware for cloud services. The proposed method detects infected files by estimating the entropy to synchronize files based on uniformity, one of the characteristics of encrypted files. For the experiment, files containing sensitive user information and system files for system operation were selected. In this study, we detected 100% of the infected files in all file formats, with no false positives or false negatives. We demonstrate that our proposed ransomware detection method was very effective compared to other existing methods. Based on the results of this paper, we expect that this detection method will not synchronize with a cloud server by detecting infected files even if the victim systems are infected with ransomware. In addition, we expect to restore the original files by backing up the files stored on the cloud server.

## 1. Introduction

Due to the fourth industrial revolution, data-based services such as cloud computing and big data have emerged. Cloud computing is a technology that processes and stores information in computing environments that provide high-level resources at a low cost [1]. Big data refers to extremely large amounts of unstructured data that have value based on their characteristics and require an analysis technique that can process a large amount of data [2]. The basis of cloud services is data, and these services cannot operate successfully if the reliability and integrity of the data are not guaranteed. Meanwhile, valuable data stored in the cloud are attractive targets; attacks can cause serious damage through ransomware attacks that threaten to manipulate data unless money is paid [3].

Ransomware restricts access by holding computer systems hostage and demanding a ransom in exchange for releasing the restrictions. Specifically, ransomware encrypts sensitive information stored on the victim system and then requires money to provide the decryption keys [4,5,6]; most of the target systems of ransomware are PCs. Investigators have tested various methods to detect ransomware and prevent system infections [7,8,9,10]. These ransomware detection methods are classified into five broad categories: based on file-based detection, system-based behavior detection, resource-based behavior detection, connection-based behavior detection, and entropy-based ransomware detection. 

File-based detection refers to the detection of signatures that perform malicious actions in system files. However, this method has two major limitations: It cannot detect new ransomware and has high false positives and false negatives. In particular, file-based detection has not yet been applied in cloud services [11]. On the other hand, system-based behavior detection involves detecting malicious behavior in systems using integrity checks and behavior blocking. This approach verifies the integrity of files and directories because ransomware encrypts files and involves tracking malicious behavior related to file access on systems. However, this method has high false-positive rates and requires a lot of verification time to verify integrity. Furthermore, system-based ransomware detection has also not been applied in cloud services [12]. 

Resource-based behavior detection detects malicious activity that causes exceptions that increase CPU and I/O usage because I/O is required for file access, and the CPU is required for encryption. However, this method requires much time to collect resource information, has high false-positive rates, and has not been addressed in cloud services [12,13]. Connection-based behavior detection attempts to connect to networks based on the characteristics of ransomware that connects to C&C servers to receive the keys needed for encryption. However, this method cannot detect any ransomware that does not connect to a network, and, as with other ransomware detection methods, it has not been applied to cloud services [14]. Finally, the entropy-based ransomware detection method is the main concept of the technology proposed in this paper and is a method for detecting files infected by ransomware based on the high entropy of ciphertexts. However, this method only calculates entropy with the general Shannon entropy information theory, and is limited since various other entropy estimation methods are not applied. In addition, this method is not considered for cloud services [15].

As discussed above, existing ransomware detection methods cannot detect infected files transmitted to cloud services. Specifically, when system data backed up to the cloud are infected with ransomware in a victim system, the backup data stored in the cloud are also infected; files encrypted on the personal computer are encrypted in the cloud storage. Thus, when a ransomware attack happens, victims cannot restore or overwrite their in-house files with their cloud backups because the cloud files are locked as well. Therefore, this paper explains a method for effectively detecting ransomware in cloud storage files to solve this problem. Furthermore, the proposed method detects whether cloud files are infected with ransomware based on their cryptographic characteristics. The contributions of this paper are as follows.

Existing ransomware detection methods cannot detect ransomware infection of files stored in the cloud. However, our method in this paper effectively detects ransomware by estimating, in reality, the entropy of files transferred to cloud servers. This allows users to secure their files stored on cloud servers and restore files in the victim system encrypted by ransomware.

Existing ransomware detection methods have low detection rates and take a long time to collect necessary information (such as resources or network connections) for detection. However, our proposed method has a high detection rate and low false positives and false negatives because we can directly detect files based on their characteristics, making it possible to efficiently detect files infected with ransomware. 

The proposed method derived an optimized entropy of files infected by ransomware for various file formats. Thus, it is possible to determine whether various file formats are infected by ransomware according to the defined threshold.

The structure of this paper is as follows. In Section 2, we propose a ransomware detection method based on the characteristics of infected files and estimation of entropy, and we describe the configuration of the detection module. In Section 3, we then describe the experimental conditions and goals, and the experimental results of detecting ransomware-infected files. Finally, Section 4 concludes our paper.

## 2. The Proposed Detection Methodology

In this section, we describe our proposed ransomware detection methodology in more detail. As mentioned in the Introduction, the existing methods for detecting ransomware, such as file-based detection, system-based behavior detection, resource-based behavior detection, and connection-based behavior detection, have distinct disadvantages—in particular, the problem that detection in cloud services is not considered. Therefore, in this paper, entropy-based ransomware detection methods are focused on in terms of effective ransomware detection and file recovery in cloud services.

**Figure 1:**
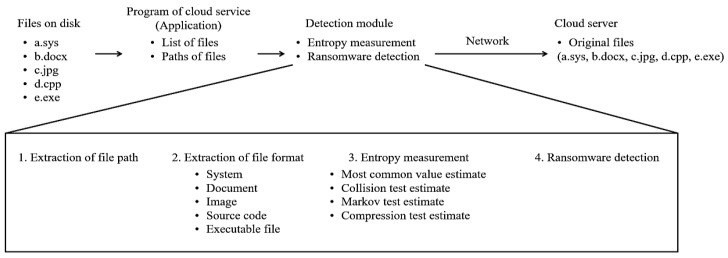
The proposed detection methodology.

The proposed ransomware detection methodology is shown in Figure 1. For detection, a detection module obtains a list of files to be transferred to the cloud server. Then, the module detects the ransomware by measuring the entropy, characteristic of encrypted files infected with ransomware. At this time, to determine whether or not to detect ransomware, the entropy change trend between the case of infection with ransomware and the case of non-infection is compared to determine the entropy threshold value for each file format. Through the entropy threshold derived in this way, files infected with ransomware are identified. Through this, eventually, infected files do not synchronize with the cloud server. The following paragraph summarizes the overall steps of the proposed detection methodology:

Step 1. Check the files stored inside a disk
Step 2. Acquire the path and list of files to be uploaded to a cloud service application
Step 3. Run the ransomware detection module
Step 4.1. Extract the path of the file to upload
Step 4.2. Extract the extension of the file to upload
Step 4.3. Extract the entropy of the file to upload
Step 4.4. Detect the ransomware-infected file
Step 5. Terminate the ransomware detection module
Step 6. Synchronize the file to the cloud service

### 2.1. Characteristics of Ransomware-Infected Files

The proposed detection method utilizes a feature that appears in ransomware-infected files that is not a feature of clean files. Ransomware generally encrypts to prevent access to files containing sensitive information. In terms of cryptography characteristics, the ciphertext generated by cryptography is statistically uniform; for example, if the value of the cipher result is from 0x00 to 0xFF, the probability of each generated cipher value should be the same [16]. However, suppose bias is applied to ciphertext, such as a specific value or a specific range of values. In that case, the problem is that it is possible to decrypt based on the probabilities of values that are generated more or less frequently [17]. To solve this problem, the cryptography technique we propose is designed so that the probability of occurrence of each value generated by the ciphertext is substantially the same. Therefore, the data in files infected with ransomware are statistically uniform because ransomware encrypts the file and the ciphertext itself is uniform. Moreover, we can detect ransomware-infected files by measuring the numbers represented by these features.

As a method for measuring uniformity, there is an entropy estimate. According to the National Institute of Standards and Technology (NIST), entropy measures disorder or randomness. For example, the uncertainty entropy for the probability (*pi*, …, *pn*) of the random variable *X* is defined as Equation (1) [18]:

(1) $$H\left(X\right)=\overset{n}{\underset{i=1}{\sum }}pi\;log\;pi$$

Based on the equation, if the encrypted data are uniform, the entropy is high. In other words, the data in a ransomware encrypted file are uniform, and the entropy of the encrypted file is higher than that of the original clean file. In this paper, we detect ransomware based on uniformity in infected files. 

### 2.2. Entropy Estimation Methods

There are various ways to measure entropy. Poisson distribution [19], Hamming distance [20], and spontaneous emission [21]. In addition, NIST provides methods and tools for measuring entropy published as NIST 800-90b [22]. The 800-90b measures randomness according to the properties of random numbers, and the measurement methods are divided into independent and identically distributed (IID) or non-IID. The IID methods are used when the generated random number is independent, whereas the non-IID methods are used when the generated ransom number is not independent [23]. In addition, the methods are classified as the statistics-based measurement method and the predictor-based measurement method. 

This paper uses statistics-based measurement methods to speed up the entropy estimate; these methods are the most common values, collision tests, Markov tests, and compression tests [24]. The most common value estimate obtains entropy by using the probability that a value will appear frequently in the input data set, shown as Equation (2). The collision test estimate defines arbitrary repetitive patterns as collisions as Equation (3) and estimates the probability of output values that appear often based on when collisions occur. The Markov test estimate measures the dependence between successive values from a set of input data as Equation (4), and the compression test estimate measures the entropy rate based on the compression capacity of the data set as Equation (3) [22].

(2) $$min-entropy=-\log_{2}\left(pu\right)$$

(3) $$min-entropy=-\log_{2}\left(p\right)\;or\;\log_{2}\left(k\right)$$

(4) $$min-entropy=-1/d\;\log_{2}\left(pmax\right)$$

### 2.3. Detection Module Configuration

**Figure 2:**
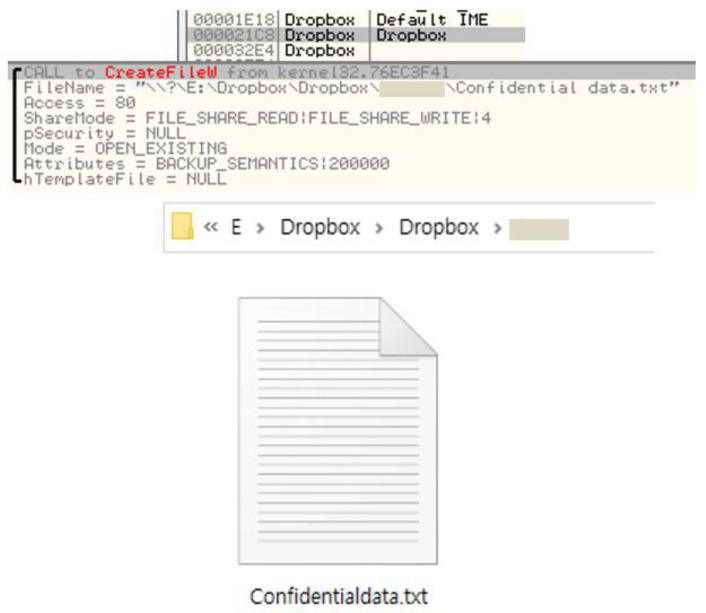
Extracting the file path synchronized to Dropbox.

The ransomware detection module proposed in this paper detects ransomware-infected files by measuring the entropy of files transferred to the cloud server. The module is located between the client software, that provides the cloud service, and the cloud server. This module needs the file path information to read the file data to measure the entropy of each file. In particular, the entropy changes according to the file format, so the module has to obtain the file format, which comprises a file that contains sensitive user information and operation files, that is, system, document, image, source code, and executable files. The list of files to be delivered to the cloud server is obtained, and then the paths of the files included in the list are extracted; finally, the file formats are obtained. Afterwards, the entropy of the file is measured, and the infected file is detected by comparing it with the threshold according to the file format. Through this process, it is possible to detect whether the victim system is infected with ransomware and detect infected files. Furthermore, if the module detects that a file transferred to the cloud server is infected by ransomware, the file will not synchronize with the uploaded file for file recovery. For this reason, the original file can be restored by downloading the uploaded file from the server. To establish the effectiveness of the detection method proposed in this paper, we experimented with Dropbox, a commercial cloud service, and verified its concept. First, the information from the file to be transferred must be extracted to obtain the file path. To extract this information, we assumed that Dropbox would need this file information for synchronization. Therefore, we expected that we would need to use the CreateFile function to extract file information. To verify this assumption, we reverse-engineered the Dropbox software and extracted the path of the synchronizing file, as shown in Figure 2. As shown in the figure, the file name to be synchronized was ‘Confidential data.txt’, which was passed as an argument to the CreateFileW function. That is, the Dropbox software calls the CreateFileW function to extract the file path. Therefore, the detection module used the hooking technique for the CreateFileW function to obtain the list of files synchronized to the Dropbox server.

The detection module is connected to Dropbox software because it does not need to be inserted into the software. That is, the detection module is implemented separately to verify the proposed concept and is connected to the Dropbox code to detect ransomware. Here, the hooking technique refers to manipulating the call flow of codes or functions.

**Figure 3:**
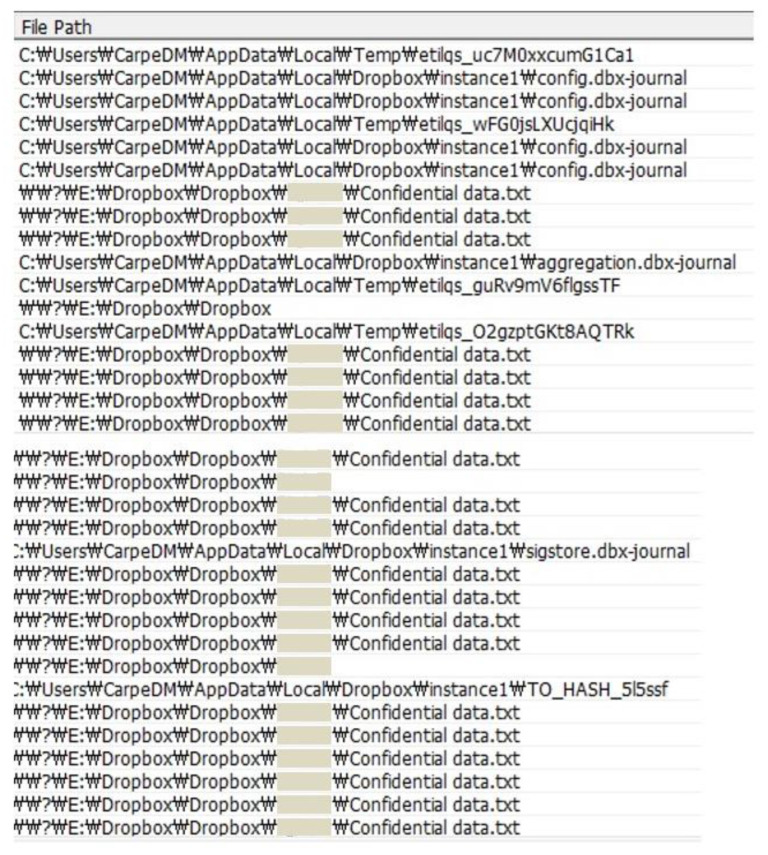
Synchronized file paths extracted from the detection module.

For example, the path of the file is obtained by compulsorily manipulating the call flow of the CreateFileW function to the detection module because the module cannot extract the list of files synchronized by Dropbox. As a result, the path of the synchronized file is obtained from the detection module that embeds to the Dropbox software using the hooking technique, and the experimental result is shown in Figure 3.

In this process, some paths are duplicated or assumed to be Dropbox files or folders. For example, there are duplicated “config.dbx-journal” files and “Dropbox” folders. In this way, unnecessary and duplicate files are removed to obtain optimized synchronized file paths and lists.

**Figure 4:**
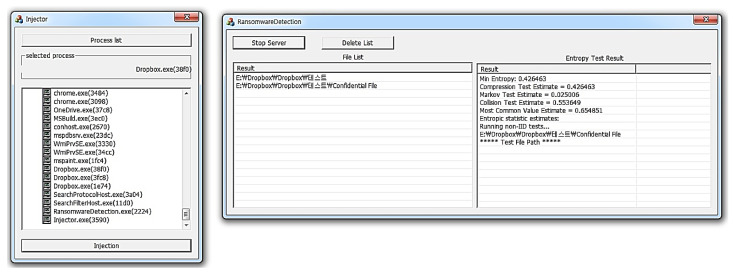
Results of the entropy estimation for a single file synchronized with Dropbox.

By extracting the file list and file path, the detection module can read the data of the files being synchronized with the Dropbox server and then detect the infected files by measuring the entropy-based on the read data. For measuring entropy, NIST provides both statistics-based and predictor-based methods. However, predictor-based measurement has the disadvantage of taking a comparatively long time, and thus it is difficult to use in measuring entropy in real-time. Therefore, we use faster, statistic-based measurement in this paper. Figure 4 shows the result of measuring the entropy of a file uploaded to the Dropbox server. 

On the right side is the detection module, which outputs a list of files to be synchronized and an entropy estimate result of the files included in the list. In the experiment, the ‘confidential file’ is synchronized with the Dropbox server. Consequently, the most common value estimate, collision test estimate, Markov test estimate, and compression test estimate were 0.654851, 0.553649, 0.025006, and 0.426463. Hence, the min-entropy was 0.426463.

## 3. Experimental Preparation and Implementation

This section describes our experimental conditions and goals, along with the experimental results of measuring the entropy of synchronized files.

### 3.1. Experimental Conditions

Based on the methodology proposed in Section 2, we verify ransomware detection by measuring the entropy of the files uploaded to the Dropbox cloud server. The specific target files are a system file, a document file, an image file, a source code file, and an executable file, which are files related to sensitive user information and system operations. Ransomware does not encrypt all files stored on a disk at once, but it also infects a single file sequentially, depending on the conditions. Based on this assumption, a sample ransomware similar to the actual ransomware was produced and tested in a way that infects the target files. For the experiment, we assumed that 10 file formats were infected with 100 files in each format. We analyzed the entropy measurement results and entropy change trends to effectively detect ransomware when 10, 20, …, 100 files were infected. Through this process, the module determined a threshold to effectively detect infection by comparing and analyzing the results of the entropy measurement for the ransomware infection and encryption processes.

This experiment was based on three goals: First, the entropy measurement results for each file format would be compared and analyzed to determine the reference values for detecting ransomware infections according to the file formats; second, to compare and analyze the entropy changes; and third, based on the two measurement results, to derive the optimal baseline for detection by analyzing the entropy change and the detection, false-positive, and false-negative rates. 

**Figure 5:**
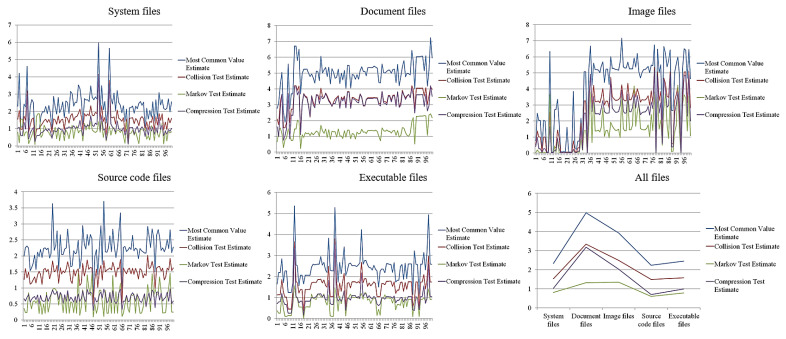
Entropy estimation results for 100 clean files per file format.

First, because measuring entropy requires reference values for each clean file format, our module measured the entropy of 100 files in each format; the result is shown in Figure 5. Second, Table 1 shows the averages of entropy for the 100 files in each format used to estimate the threshold of each format for clean files. 

As the figure shows, the entropy results change for some but not all formats of clean files; specifically, 4, 7, 10, 3, and 4 high peaks corresponded, respectively, to system, document, image source code, and executable files. That is, some but not all clean files showed high entropy. By amount, respectively, fewer than 6, 8, 8, 4, and 6, system, document, image, source code, and executable files showed entropy. By average, more than 3, 5, 4, 3, and 3, the system, document, image, source code, and executable files, respectively, showed entropy.

As shown in Table 1, the entropy averages for the 100 clean files in each file format differed according to the measurement methods; for example, the most common value estimate of entropy for the system files was 2.32, but the Markov test estimate was 0.81. Most of the most common value estimates of entropy were high, and the Markov test estimates were low. Therefore, we concluded that the optimal reference value would change according to the measurement methods and derived reference values to detect ransomware infection from each of the four methods. We found that the system, source code, and executable files had similar entropy results; in particular, entropy was lowest among the source code files and highest among the document files. Source code files are statistically biased, but image files reflect the statistical tendency of the data distribution to be small.

Therefore, we derived the threshold for detecting ransomware-infected files based on the average and peak entropy, which occurs as a high peak in clean files.

### 3.2. Comparison and Analysis of Entropy by File Format

**Figure 6:**
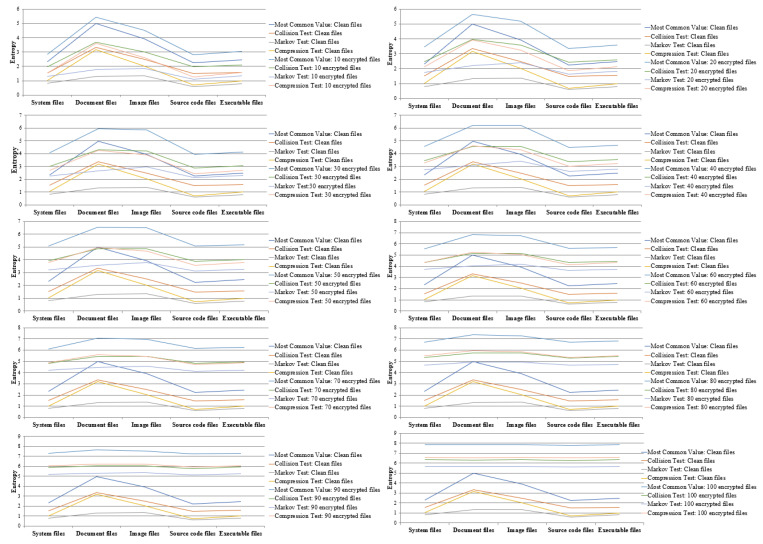
Results of the entropy estimate per 10 ransomware-infected files.

Entropy differed by file format, as discussed in Section 3.1. For this reason, detecting ransomware-infected files requires obtaining the file formats and the reference values for each format. This paper measured the entropy by file format, assuming that one of every 10 clean files is infected by ransomware. We present the average entropy measurements for 100 files in Figure 6. 

As shown in the figure, the results show that the entropy increased with the number of infected files; in particular, if more than 90 files were infected, the entropy was higher in the infected files with all measurement methods than it was in the clean files. This means that all methods could detect infected files. On the contrary, if 50 or fewer files are infected, it is hard to distinguish them because the entropy of infected files is similar to the entropy of clean files. In particular, if more than 60 files were infected, the files in all formats had a constant average entropy without significant differences. This means that the entropy in more than 60 ransomware-infected files can be a reference value.

To detect ransomware-infected files and derive the reference value, the value is determined to be the least to distinguish between infected and clean files. In terms of results for each file format and measurement methods, the number of the system file, document, image, source code, and executable files were 30 files with the most common value estimate, 40 files with collision test estimate, 30 files with Markov test estimate, and 30 files with compression test estimate. These can be determined as the average entropy values for the reference when at least 40 files are infected.

To distinguish the numerical entropy values more clearly, we present in Table 2 all the entropy values measured by file format and measurement method and the highest entropy value for clean files. In the Table, Most, Collision, Markov, Compression, C, I, and P are denoted most common value estimate, collision test estimate, Markov test estimate, compression test estimate, clean files, infected files, and peaked entropy of clean files, respectively.

The results for each file format are as follows. System files are detected when 30 files are infected with the Markov test estimate, and document files are detected when 30 files are infected with the collision test estimate. Image files are detected when 70 files are infected with collision test estimate, Markov test estimate, and compression test estimate. Finally, source code files are detected when 10 files are infected with compression test estimate, and executable files are detected when 20 files are infected with Markov test estimate. Based on these results, the infection could be detected among the fewest infected source code files, and image files could be detected when most were infected. Therefore, we consider that effectively detecting ransomware-infected files requires selecting the optimal method for measuring the entropy for each file format.

### 3.3. Comparison and Analysis of Entropy Changes by Number of Infected Files

**Figure 7:**
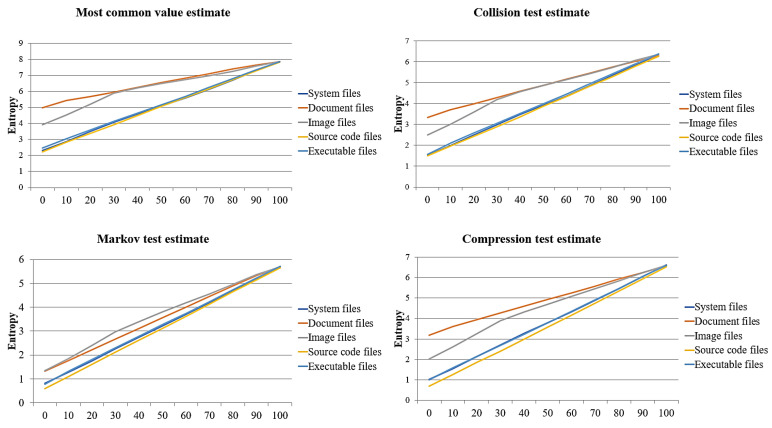
Changes in the entropy estimate per 10 infected files by the estimate method.

The results shown in Figure 6 and Table 2 indicate that the entropy increases as the number of infected files increases. Therefore, reference values are required to detect a specific minimum number of ransomware-infected files. To determine these reference values, we compared and analyzed the changes in entropy according to the number of infections. For this paper, we assumed that 100 files were infected with ransomware per 10 files and derived the changes in entropy by the number of infections. The result is shown in Figure 7.

The results showed that the file formats with the highest entropy according to the most common value estimation, collision test estimation, Markov test estimation, and compression test estimation were document, document, image, and document files. Source code files had the lowest entropy. According to the trend of change, the most common value estimate was the measurement method with the sharpest change by the number of infected files. The method with the slightest change was the collision test estimation. The file formats with sharp changes were system, source code, and executable files, while the document files showed the slightest changes. To summarize these results, our proposed detection module was likely to detect ransomware according to the number of infections when the entropy changes abruptly. That is, when the entropy was measured using the most common value estimates for system files, source code files, and executable files, we could effectively detect ransomware in a cloud environment.

On the analysis of the points of gradual changes by file format, the optimal number of infected files for the most common value and collision, Markov, and compression test estimates was 30 files, similar to the results shown in Figure 6 and Table 2. For this reason, we consider that with a threshold of 30 files, ransomware will be effectively detected. In this paper, we derived optimal reference values by analyzing the changes in entropy using the measurement method and determined a reference value to effectively detect ransomware based on the detection rate.

**Figure 8:**
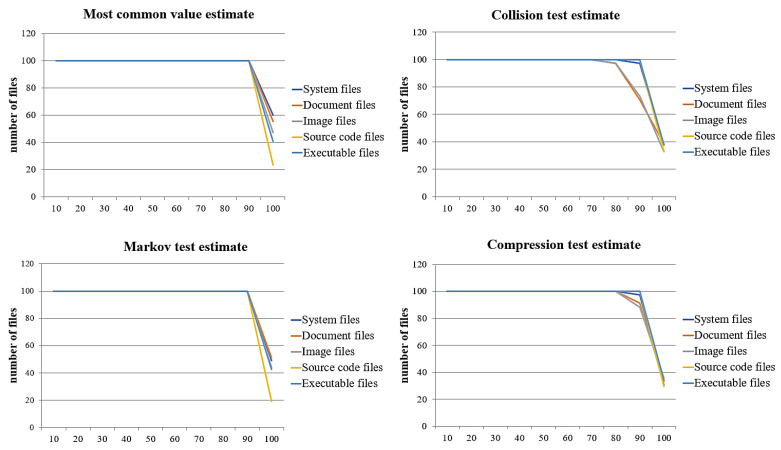
Average detection rate based on entropy average per 10 files infected by ransomware.

We analyzed the detection rate according to the number of infections by file format, assuming that in every 100 files, 10 files are infected by ransomware. The result is shown in Figure 8.

All measurement methods detected 100% of the ransomware-infected files using the average entropy for 70 infected files. However, using the entropy average for 80 infected files, the detection rate for the collision test estimate was lower than that for the other measurement methods. In addition, the detection rate decreased sharply for all measurement methods using the average entropy of 100 infected files. In particular, the compression test estimate had the lowest detection rate, whereas the most common value estimate had the highest rate. By file format, using the average entropy of 80 infected document and image files, we identified several ransomware-infected files that could not be detected. However, our proposed method detected all ransomware-infected source code and executable files using the average entropy of 90 infected files.

The results in the previous two sections show that the detection rate was high using the average entropy of 30 or 40 infected files. However, we found that the detection rate was as high as 100% using all the average entropies from 10 to 70 infected files; this suggests that the average entropy can be used extremely efficiently as a reference value. In contrast, some reference values showed high false-positive and false-negative rates even with high detection rates. As shown in Table 2, some files in each format had larger than average entropy. These were cases of false positives, and for this paper we derived the optimal reference values to minimize false positives and false negatives.

### 3.4. Determining of Optimal Baseline Values by Detection Rates, False-Positive Rates, and False-Negative Rates

**Figure 9:**
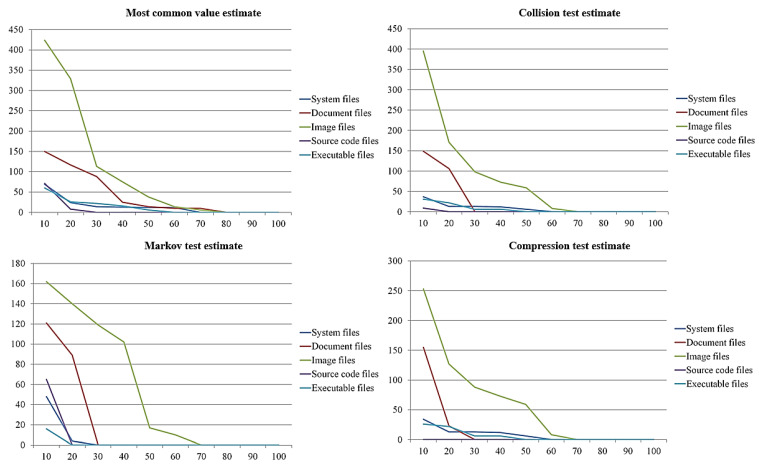
Total number of false positive files.

This paper derives optimal baseline values for detecting ransomware-infected files based on the above entropy comparison and analysis results, detection rates, false positives, and false negatives. Because these rates differed by measurement method, we determined the optimal baseline values using this method. First, Figure 9 shows the false-positive rates by the measurement method.

The results show that all measurement methods had low false-positive rates with a large number of infected files. With an average entropy of 70 infected files, most methods had very low false-positive rates. By the measurement method, the Markov test estimate had a low false-positive rate during the longest interval, and the most common value estimate had a low false-positive rate during the shortest interval. By file format, the source code files had the lowest false-positive rate in the longest interval in all measurement methods, and the image files had the lowest rate in the shortest interval. By measurement method and file format, the most common values, collision test, and compression test estimates had low false-positive rates with the source code files for the longest interval. The Markov test estimate had a low false-positive rate for the longest interval with the executable and source code files. Moreover, the most common value estimate had a low false-positive rate with the shortest interval in document files. The Markov, collision, and compression tests estimates had low false-positive rates in the shortest interval with the image files.

By the number of files in which a false-positive occurred, the image files had the false positives, and the source code had the fewest with all measurement methods. For the number of files with false positives by the measurement method, the Markov test estimate had the fewest false positives, and the most common value estimate had the false positives.

**Figure 10:**
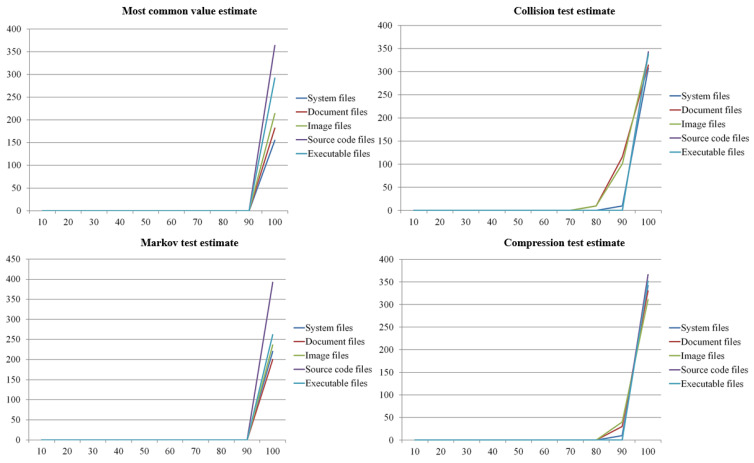
Total number of true-negatives files.

As with the false-positive rate, to detect ransomware efficiently, we derived the baseline entropy with the optimal false-negative rate, while the rates by the measurement method are shown in Figure 10. Again, all measurement methods had low false-negative rates with the average entropy for a low number of infected files. The average entropy for most of the 70 infected files had a very low false-negative rate.

By both the measurement method and the file format, the source code files had a low false-negative rate in the longest interval, and the document and image files had low rates in the shortest interval. The source code files had a low false-negative rate for the longest interval, and document and image files had low false-negative rates with the shortest interval. The most common value estimate and the Markov test estimate had low false-negative rates for the longest intervals in all file formats by combined measurement method and file format.

The number of files in which a false negative occurred, source code files had the most false negatives, and system files had the fewest. The most common value estimate had the fewest false negatives and the collision test estimate the most by the measurement method.

Based on the above results for false positives and false negatives, we derived baseline values to detect ransomware-infected files. We determined the baseline values as the entropy intervals with the fewest false positives and false negatives and the highest detection rate, as shown in Table 3.

We found baseline entropy values with zero false positives and false negatives and 100% detection in all measurement methods. Image files gave the shortest interval for the baseline entropy values by file format, and source code files had the longest interval.

The most common value estimate by each measurement method was that document files had the shortest interval at 0.25 and source code files had the longest interval at 3.35. In the collision test estimate, image files had the shortest interval at 0, and source code files had the longest interval at 3.36. In the Markov test estimate, the image files had the shortest interval at 0.8, and the source code files had the longest interval at 3.52. Finally, in the compression test estimate, image files had the shortest interval at 0.38, and source code files had the longest at 4.66. Therefore, the ransomware detection method based on entropy proposed in this paper is effective and completely accurate.

## 4. Conclusions

This paper proposed a method to effectively detect ransomware by measuring the entropy of files stored on a cloud server. The idea of the proposed detection method is uniformity, one of the characteristics of ransomware-infected encrypted files; that is, the probability that each value included in the data range for the encrypted files is almost the same. Therefore, we measured the uniformity of the files synchronized to the cloud server based on entropy. For the experiment, we collected system, document, image, source code, and executable files, which are the files necessary for system operation and include sensitive user information. We estimate the entropy by comparing the entropy of ransomware-infected encrypted files with that of clean files. To derive the baseline entropy for infected files, we compared and analyzed the entropy by file format and number of ransomware infections; based on these results, we derived the optimal baseline values for detecting ransomware-infected files. The baseline value we derived detected 100% of all ransomware-infected files, which means 0% false positives and false negatives. Therefore, the suggestions in this paper detect ransomware using ransomware-infected files very effectively. Furthermore, this allows one to recover files stored on a cloud server by backing up files stored on the cloud server.

The limitations of the measures proposed in this paper are as follows: First, it was assumed that ransomware infects the victim system files slowly and sequentially; the type of file was tested for only system files, document files, image files, source code files, and executable files. Second, the entropy characteristics of encrypted files were derived based on sample ransomware, which is very similar to actual ransomware, but not actual ransomware. Third, encryption and compression have essentially similar characteristics. Namely, after compression, the entropy of the file is increased. For this reason, the proposed method has the limitation that it is difficult to distinguish compressed files from ransomware-infected files. Fourth, the proposed method is to detect ransomware through entropy measurements. Therefore, we focused on general ransomware, and did not consider ransomware variants, such as partial intermittent encryption, which does not have a large change in entropy of files. Nevertheless, we consider that even ransomware that utilizes partial encryption can be detected if it partially measures entropy. Moreover, according to “The inadequacy of entropy-based ransomware detection”, the inadequacy of the entropy-based ransomware detection method was asserted by normalizing entropy with a base 64 encoding technique [25]. However, if a base 64 encoded file is identified from the ransomware defender’s point of view, entropy-based ransomware detection can detect ransomware-infected files by measuring the entropy of the files after decoding. Finally, the criteria for ransomware infected files were derived assuming that the format of the target file was known, and it is believed that of the entropy measurement methods, only the Markov test estimation could be applied.

In the future, we will consider these limitations and study ways to detect external disks, USB storage devices, secure disks, and ransomware on secure USB to backup or store files and cloud services. Moreover, to verify the applicability of the proposed methodology in the real world, the file format will be expanded, and the publicly available ransomware sample data will be configured for verification.

## Figures and Tables

**Table 1:** Entropy averages for 100 files in each file format.

File Format	Most Common Value Estimate	Collision Test Estimate	Markov Test Estimate	Compression Test Estimate
System files	2.32600693	1.52923478	0.81051535	1.0281958
Document files	4.97830862	3.34043146	1.31800652	3.17635457
Image files	3.91966737	2.49202631	1.34891675	2.0287627
Source code files	2.23608321	1.48598446	0.59899854	0.69851822
Executable files	2.45548063	1.56868541	0.78475101	0.99562768

**Table 2:** Results of the comparison entropy estimate per 10 ransomware-infected files with clean files.

Number of Infected Files		System Files			Document Files			Image Files			Source Code Files			Executable Files		
		C	I	P	C	I	P	C	I	P	C	I	P	C	I	P
10 files	Most	2.32	2.86	5.97	4.97	5.41	7.23	3.91	4.51	7.16	2.23	2.81	3.70	2.45	3.04	5.36
	Collision	1.52	1.97	4.15	3.34	3.69	4.20	2.49	3.00	5.34	1.48	1.96	2.02	1.56	2.10	3.80
	Markov	0.81	1.28	1.85	1.31	1.76	2.42	1.34	1.85	4.23	0.59	1.10	1.45	0.78	1.32	1.57
	Compression	1.02	1.55	4.08	3.17	3.61	4.19	2.02	2.61	5.13	0.69	**1.26**	0.96	0.99	1.58	3.52
20 files	Most	2.32	3.48	5.97	4.97	5.65	7.23	3.91	5.18	7.16	2.23	3.36	3.70	2.45	3.59	5.36
	Collision	1.52	2.48	4.15	3.34	3.97	4.20	2.49	3.59	5.34	1.48	**2.42**	2.02	1.56	2.58	3.80
	Markov	0.81	1.75	1.85	1.31	2.20	2.42	1.34	2.40	4.23	0.59	**1.61**	1.45	0.78	**1.81**	1.57
	Compression	1.02	2.13	4.08	3.17	3.93	4.19	2.02	3.24	5.13	0.69	**1.84**	0.96	0.99	2.13	3.52
30 files	Most	2.32	4.05	5.97	4.97	5.94	7.23	3.91	5.86	7.16	2.23	**3.91**	3.70	2.45	4.13	5.36
	Collision	1.52	2.99	4.15	3.34	**4.27**	4.20	2.49	4.19	5.34	1.48	**2.88**	2.02	1.56	3.05	3.80
	Markov	0.81	**2.24**	1.85	1.31	2.65	2.42	1.34	2.96	4.23	0.59	**2.10**	1.45	0.78	**2.29**	1.57
	Compression	1.02	2.71	4.08	3.17	4.27	4.19	2.02	3.88	5.13	0.69	**2.40**	0.96	0.99	2.68	3.52
40 files	Most	2.32	4.58	5.97	4.97	6.22	7.23	3.91	6.22	7.16	2.23	**4.49**	3.70	2.45	4.63	5.36
	Collision	1.52	3.46	4.15	3.34	**4.57**	4.20	2.49	4.56	5.34	1.48	**3.35**	2.02	1.56	3.51	3.80
	Markov	0.81	**2.74**	1.85	1.31	3.10	2.42	1.34	3.38	4.23	0.59	**2.61**	1.45	0.78	**2.79**	1.57
	Compression	1.02	3.27	4.08	3.17	4.60	4.19	2.02	4.31	5.13	0.69	**2.98**	0.96	0.99	3.22	3.52
50 files	Most	2.32	5.09	5.97	4.97	6.53	7.23	3.91	6.48	7.16	2.23	**5.06**	3.70	2.45	5.16	5.36
	Collision	1.52	3.92	4.15	3.34	**4.86**	4.20	2.49	4.85	5.34	1.48	**3.86**	2.02	1.56	**3.99**	3.80
	Markov	0.81	**3.21**	1.85	1.31	3.56	2.42	1.34	3.80	4.23	0.59	**3.11**	1.45	0.78	**3.26**	1.57
	Compression	1.02	3.80	4.08	3.17	4.93	4.19	2.02	4.69	5.13	0.69	**3.57**	0.96	0.99	**3.78**	3.52
60 files	Most	2.32	5.57	5.97	4.97	6.81	7.23	3.91	6.71	7.16	2.23	**5.59**	3.70	2.45	**5.67**	5.36
	Collision	1.52	**4.35**	4.15	3.34	**5.15**	4.20	2.49	5.14	5.34	1.48	**4.33**	2.02	1.56	**4.45**	3.80
	Markov	0.81	**3.69**	1.85	1.31	4.00	2.42	1.34	4.19	4.23	0.59	**3.61**	1.45	0.78	**3.73**	1.57
	Compression	1.02	**4.32**	4.08	3.17	5.25	4.19	2.02	5.06	5.13	0.69	**4.15**	0.96	0.99	**4.33**	3.52
70 files	Most	2.32	**6.12**	5.97	4.97	7.08	7.23	3.91	6.97	7.16	2.23	**6.15**	3.70	2.45	**6.23**	5.36
	Collision	1.52	**4.83**	4.15	3.34	**5.45**	4.20	2.49	**5.43**	5.34	1.48	**4.81**	2.02	1.56	**4.93**	3.80
	Markov	0.81	**4.19**	1.85	1.31	4.45	2.42	1.34	**4.55**	4.23	0.59	**4.13**	1.45	0.78	**4.23**	1.57
	Compression	1.02	**4.88**	4.08	3.17	5.58	4.19	2.02	**5.45**	5.13	0.69	**4.74**	0.96	0.99	**4.90**	3.52
80 files	Most	2.32	**6.70**	5.97	4.97	**7.37**	7.23	3.91	**7.25**	7.16	2.23	**6.71**	3.70	2.45	**6.78**	5.36
	Collision	1.52	**5.34**	4.15	3.34	**5.75**	4.20	2.49	**5.72**	5.34	1.48	**5.29**	2.02	1.56	**5.41**	3.80
	Markov	0.81	**4.69**	1.85	1.31	4.90	2.42	1.34	**4.94**	4.23	0.59	**4.64**	1.45	0.78	**4.72**	1.57
	Compression	1.02	**5.46**	4.08	3.17	5.92	4.19	2.02	**5.83**	5.13	0.69	**5.33**	0.96	0.99	**5.47**	3.52
90 files	Most	2.32	**7.28**	5.97	4.97	**7.62**	7.23	3.91	**7.55**	7.16	2.23	**7.26**	3.70	2.45	**7.33**	5.36
	Collision	1.52	**5.85**	4.15	3.34	**6.02**	4.20	2.49	**6.07**	5.34	1.48	**5.78**	2.02	1.56	**5.91**	3.80
	Markov	0.81	**5.18**	1.85	1.31	5.33	2.42	1.34	**5.35**	4.23	0.59	**5.13**	1.45	0.78	**5.21**	1.57
	Compression	1.02	**6.04**	4.08	3.17	6.23	4.19	2.02	**6.23**	5.13	0.69	**5.92**	0.96	0.99	**6.05**	3.52
100 files	Most	2.32	**7.83**	5.97	4.97	**7.83**	7.23	3.91	**7.83**	7.16	2.23	**7.81**	3.70	2.45	**7.84**	5.36
	Collision	1.52	**6.34**	4.15	3.34	**6.28**	4.20	2.49	**6.34**	5.34	1.48	**6.26**	2.02	1.56	**6.38**	3.80
	Markov	0.81	**5.69**	1.85	1.31	5.68	2.42	1.34	**5.69**	4.23	0.59	**5.64**	1.45	0.78	**5.69**	1.57
	Compression	1.02	**6.61**	4.08	3.17	6.56	4.19	2.02	**6.58**	5.13	0.69	**6.53**	0.96	0.99	**6.60**	3.52

The numbers in red are entropy values that are not between C and P. This value is used as a threshold for detecting ransomware.

**Table 3:** Optimal entropy baseline values.

Estimate Methods		System Files	Document Files	Image Files	Source Code Files	Executable Files
Most	Min	6.12	7.37	7.25	3.91	5.67
	Max	7.28	7.62	7.55	7.26	7.33
Coll.	Min	4.35	4.24	5.43	2.42	3.99
	Max	5.34	5.45	5.43	5.78	5.91
Markov	Min	2.24	2.65	4.55	1.61	1.81
	Max	5.18	5.33	5.35	5.13	5.21
Comp.	Min	4.32	4.27	5.45	1.26	3.78
	Max	5.46	5.92	5.83	5.92	6.05

## Data Availability

The data are contained within the article.
